# Association between healthy plant-based diet-lifestyle (hPDI-Lifestyle) score and incidence of coronary heart disease, and effect modification by genetic predisposition: a prospective analysis in a population-based cohort

**DOI:** 10.1016/j.lanepe.2026.101619

**Published:** 2026-02-19

**Authors:** Xiang Jun Wang, Trudy Voortman, Daniel Bos, Maryam Kavousi, Mohsen Ghanbari, Nathalie Conrad, Miranda T. Schram, Marinka Steur

**Affiliations:** a Department of Epidemiology, Erasmus MC, University Medical Centre Rotterdam, PO Box 2040, 3000 CA, Rotterdam, the Netherlands; b Deep Medicine, Nuffield Department of Women's and Reproductive Health, University of Oxford, Oxford, UK; c Department of Radiology and Nuclear Medicine, Erasmus Medical Center, Rotterdam, the Netherlands; d Department of Cardiovascular Sciences, KU Leuven, 3000, Leuven, Belgium; e Department of Internal Medicine, School for Cardiovascular Diseases, Faculty of Health, Medicine and Life Sciences, Maastricht University, Maastricht, the Netherlands; f Division of Human Nutrition and Health, Wageningen University, P.O. Box 17, 6700 AA, Wageningen, the Netherlands; g Heart & Vascular Center, Maastricht University Medical Centre, Maastricht, the Netherlands

**Keywords:** Healthy plant-based diet index, Plant-based eating, Coronary heart diseases, Cardiovascular health, Epidemiology

## Abstract

**Background:**

Healthy plant-based diet has been shown to benefit cardiovascular health and prevent coronary heart disease (CHD). However, association in combination with other ideal health behaviours on CHD prevention has been understudied. Furthermore, limited attention has been given to potential interactions with genetic CHD predisposition, which may add personalized health behaviour recommendations. We evaluated the association between healthy lifestyle and CHD incidence and investigated potential effect modification with genetically determined CHD risk.

**Methods:**

We analysed 7764 participants (mean age 63, 60.1% women) from the population-based Rotterdam Study. The degree of adherence to the healthy lifestyle was quantified by a healthy plant-based diet-lifestyle (hPDI-Lifestyle) score. Multivariable Cox proportional hazard models were used to estimate hazard ratios (HRs) and 95%-confidence intervals (CIs) for CHD according to the hPDI-Lifestyle score, stratified by polygenic risk score of coronary artery disease.

**Findings:**

We documented 918 CHD cases during 116,324 person-years of follow-up. Ideal adherence to the hPDI-Lifestyle was associated with a 20% lower CHD risk among participants at low genetic risk (HR 0.80, 95% CI 0.71–0.90), and a 44% lower CHD risk among those at high genetic risk (HR 0.56, 95% CI 0.49–0.64) compared with participants at high genetic risk but with poor adherence to the hPDI-Lifestyle (*p* for interaction <0.001).

**Interpretation:**

Our findings support recommendations to adopt a healthful plant-based diet in combination with lifestyle (non-smoking, adequate physical activity and moderate sleep duration) for personalized CHD prevention. Potential differences by genetic predisposition of lifestyle on CHD prevention warrants further investigations.

**Funding:**

This work was supported by the Erasmus Medical Centre and Erasmus University, Rotterdam, Netherlands.

**Research in context**

**Evidence before this study**

We searched PubMed from inception to January 2026 for studies examining the combined role of genetic predisposition and lifestyle behaviours in coronary heart disease (CHD) based on the following query syntax: ((genetic predisposition [Title/Abstract]) OR (genetic risk [Title/Abstract]) OR (polygenic risk score [Title/Abstract])) AND ((“Life's Essential” [Title/Abstract]) OR (lifestyle [Title/Abstract]) OR (lifestyles [Title/Abstract]) OR (behaviours [Title/Abstract])) AND ((ischemic heart disease [Title/Abstract]) OR (coronary heart disease [Title/Abstract]) OR (coronary disease [Title/Abstract]) OR (myocardial infarction [Title/Abstract]) OR (cardiovascular disease [Title/Abstract])). We considered prospective cohort studies and meta-analyses without language restrictions. Previous large-scale studies, including those from the UK Biobank and U.S. cohorts, demonstrated that compared with a favourable lifestyle, an unfavourable lifestyle was associated with a higher risk of incident coronary events among participants at high genetic risk than those at low genetic risk. However, prior studies evaluating Life's Essential 8 have largely defined healthy dietary patterns based on limited dietary components instead. Besides, most evidence comes from non-European continental populations, and long-term data in well-characterized European cohorts remain limited. Moreover, a recent GWAS for CAD, comprising the largest sample size to date, has identified novel loci 10 beyond those reported in previous studies. Thus, it underscores the importance of reevaluating the association between lifestyle factors-incorporating a healthy dietary pattern comprising more commonly consumed healthy food groups- and CHD events across populations with varying genetic predispositions, incorporating the latest GWAS finding.

**Added value of this study**

In this population-based European cohort with long-term follow-up, we investigate the joint associations of multiple healthy lifestyle factors with CHD incidence, and the interplay between genetic predisposition and lifestyle on CHD. By applying a validated lifestyle score that incorporated a healthy plant-based diet in combination with non-smoking, adequate physical activity and moderate sleep duration, alongside polygenetic risk score derived from the latest GWAS, we provide robust evidence for personalized CHD prevention. Our findings confirm and extend prior results by showing that individuals at high genetic risk was associated with prominent risk reduction through adherence to a healthy lifestyle, supporting the applicability of lifestyle interventions in European populations.

**Implications of all the available evidence**

Taken together with existing studies, our results reinforce the critical role of healthy lifestyle adherence in CHD prevention, irrespective of genetic predisposition. These findings support prioritization of lifestyle promotion within public health strategies as an equitable and effective approach to reduce CHD burden at the population level. Future research should investigate how genetic risk profiling can be integrated into precision prevention frameworks, and whether the benefits of lifestyle interventions differ across levels of genetic susceptibility.

## Introduction

Life's Essential 8 was constructed to catalyse ongoing efforts to optimize and preserve cardiovascular health (CVH) at both individual and population levels.^1^, ^2^, ^3^ The health behaviours components of Life's Essential 8 include diet, physical activity, nicotine exposure, and sleep health.^2^ The three non-dietary behaviours–physical activity, smoking, and sleep—are key modifiable lifestyle factors with well-established links to cardiovascular health and are known to interact synergistically with diet in shaping overall cardiometabolic risk.^4^, ^5^, ^6^ Therefore, integrating these behaviours with diet enables a more comprehensive assessment of overall lifestyle quality and its impact on cardiovascular outcomes. Among dietary recommendations, Dietary Approaches to Stop Hypertension (DASH)- and Mediterranean-style eating patterns are considered as most conducive to achieving optimal CVH.^7^, ^8^, ^9^ A healthy plant-based diet, which is generally similar to a Mediterranean-style dietary pattern, but specifically emphasizing higher intake of healthy plant-based foods (e.g., fruits, vegetables, whole grains, nuts, olive oil and coffee), and lower intake of unhealthy plant-based foods (e.g., refined cereals, sugary drinks and pastries) and animal-based foods, has also been largely reported to be beneficial for CVH.^10^ Beyond its cardiometabolic benefits, a healthy plant-based diet is also more environmentally sustainable, as it reduces reliance on animal-based foods, lowers greenhouse gas emissions, irrigation water, nitrogenous fertilizer and high-quality cropland needs, and promotes more resource-efficient food production.^11^^,^^12^ However, no studies to date have investigated the effect of plant-based diet in combination with other health behaviours for prevention of coronary heart disease (CHD).

Large-scale genome-wide association studies (GWAS) have identified numerous genetic variants associated with coronary artery disease (CAD) risk, enabling the development of polygenic risk scores (PRS).^13^^,^^14^ Previous evidence showed genetic and lifestyle factors were independently associated with incident CHD.^7^^,^^8^ However, they also found that as compared with a favourable lifestyle, an unfavourable lifestyle was associated with a higher risk of incident coronary events among participants at high genetic risk than those at low genetic risk.^7^^,^^8^ Additionally, a recent GWAS for CAD, comprising the largest sample size to date, has identified novel loci^15^ beyond those reported in previous studies.^7^^,^^16^ Compared to PRS developed in 2015,^16^ the updated 2022 PRS demonstrated a stronger association with incident CAD and an enhanced more accurate risk stratification.^15^ This underscores the importance of reevaluating the association between lifestyle factors and CHD events across populations with varying genetic predispositions, incorporating the latest GWAS finding.

To advance the knowledge of the role of a healthy plant-based diet in combination with other health behaviours components of Life's Essential 8 on CHD prevention and provide evidence on formulating personalized health behaviours recommendations, this study aimed to investigate (1) the associations of a healthy plant-based diet combined with other lifestyle factors (smoking, physical activity and sleep) with CHD incidence and (2) whether these associations vary based on individual's genetic predisposition to CHD.

## Methods

### Study design and subjects

This study was carried out using data from sub-cohorts of the Rotterdam Study, a population-based prospective cohort study of adults aged ≥45 years living in the well-defined district of Ommoord in Rotterdam, the Netherlands. Details on the study design have been described elsewhere.^17^ Briefly, the first sub-cohort (RS-I) started in the period of 1989–1993 and recruited 7983 participants aged ≥55 years. A second sub-cohort (RS-II) started in 2000–2001 enrolling 3011 individuals who turned ≥55 years or moved into the study district since the start of the study. The RS was further enlarged with a third sub-cohort (RS-III) and a fourth sub-cohort (RS-IV), in 2006 with 3932 participants aged ≥45 years and in 2016 with 3005 persons aged ≥40 years. By the start of 2021, 17,931 participants were included in total. At enrolment, participants were interviewed at home and underwent a series of examinations in the research centre. Subsequent follow-up visits take place every 3–6 years. The Rotterdam Study has been approved by the Medical Ethics Committee of Erasmus Medical Centre (registration number MEC 02.1015) and by the Netherlands Ministry of Health, Welfare and Sports (License number 1071272-159521-PG). Informed consent was obtained from all participants.

For the prospective analysis of incident CHD, an original total of 14,926 participants at baseline from RS-I, RS-II, and RS-III were included. Eligibility criteria for inclusion were: (i) complete and valid dietary data, defined as an estimated energy intake between 500 and 5000 kcal/day; (ii) availability of genetic data for calculating the PRS for CAD; and (iii) provision of informed consent along with valid follow-up information on CHD incidence. Participants with complete and valid data on dietary intake and genetic variants associated with CAD were included in the analyses. In both set of analyses, individuals with pre-existing CHD at baseline were excluded.

### Dietary assessment and construction of healthy plant-based diet index

Baseline dietary intake was assessed using semi-quantitative food frequency questionnaires (FFQs) followed by an interview with a trained dietician at the research center.^18^^,^^19^ The FFQs have been validated against repeated food records, dietary histories, 24 h urinary urea and biomarker measurements, demonstrating that participants can be adequately ranked according to their food and nutrient intake.^18^ Food intake data were utilized to estimate participants’ daily energy and nutrient intake levels by mapping food items to the Dutch Food Composition Table (NEVO),^20^ and multiplying their nutrient content with the frequency and quantity of consumption.

Based on the dietary data, a healthy plant-based diet index (hPDI) was calculated based on methods reported previously.^21^ Briefly, food items measured by FFQs were categorized into 22 food groups as shown in Supplemental Table S1. These 22 food groups were further classified into three categories: (i) a healthy plant-based food category (7 healthy plant-based food groups: fruits, vegetables, whole grains, nuts, legumes, tea and coffee, and vegetable oils); (ii) an unhealthy plant-based food category (4 unhealthy plant-based food groups: refined grains, potatoes, sugary beverages, and sweets) and (iii) an animal-based food category (11 animal-based food groups: low-fat milk, low-fat yogurt, full-fat milk, full-fat yogurt, cheese, fish, eggs, animal fat, unprocessed white meat, processed and red meat, dessert and sugary dairy). Margarine intake was excluded from the hPDI, but included as a covariate in the analyses models, because of the substantial reduction in trans fatty acids in margarines in the Netherlands since 1980, driven by evidence linking trans-fats to increased CHD risk,^22^ and aligns with previous study's approach.^23^ Mixed foods, such as pizza, soup, pancake and spring rolls, were categorized into a miscellaneous food group and included as a covariate. Alcoholic beverage was excluded from the hPDI due to its distinct and specific associations with various health outcomes.

We constructed an hPDI by ‘positively’ scoring participants' intake levels of each healthy plant-based food group to reflecting diets containing relatively more healthy plant-based foods, emphasizing the quality of plant-based foods, with a score of 1 to participants with the lowest intake levels and a score of 5 to participants with the highest intake levels. By contrast, participants' intake levels of unhealthy plant-based food groups and animal-based food groups were scored ‘reversely’, with a score of 1 indicating the highest intake levels and a score of 5 indicating the lowest intake levels of each unhealthy plant-based and animal-based food groups. Participants' scores for each of the 22 food groups were summed together, yielding a theoretical range from 22 to 110 (Supplemental Table S1). A score of 22 corresponded to the lowest intake of healthy plant-based foods and the highest intake of unhealthy plant-based and animal-based foods, while a score of 110 indicated the highest intake of healthy plant-based foods and the lowest intake of unhealthy plant-based and animal-based foods.

### Assessment of non-dietary health behaviours and construction of a hPDI-lifestyle score

Information on smoking status and sleep duration was obtained from questionnaires during home interviews. Data on physical activity were obtained through the adapted version of the Zutphen Physical Activity Questionnaire and the LASA Physical Activity Questionnaire.^24^^,^^25^ We assigned metabolic equivalent of task (MET) scores to all activities based on the 2011 updated version of the Compendium of Physical Activities. To quantify total physical activity, we calculated MET-hours per week by multiplying the MET values of specific activities by the time (in hours per week) spent on each activity.^26^ Sleep duration was evaluated using questions, e.g., “How long did you usually sleep per night (in hours)?”.^27^

We constructed a hPDI-Lifestyle score incorporating four health behaviour components from Life's Essential 8 metrics: adherence to a healthy diet, physical activity, smoking status and sleep health (Supplemental Table S2).^2^ The detailed scoring method and rational were provided in the Supplemental Methods and Supplemental Table S2. In this study, for the metric ‘healthy diet’ we used the healthy plant-based diet index. Each component was scored on a scale of 0–100 points, and the total score was calculated by summing the component scores and dividing by four. This yielded a theoretical total score range of 0–100 points, with 100 points reflecting the greatest level of adherence to a ‘hPDI-Lifestyle’.

### Calculation of PRS of coronary artery disease

Fasting blood samples were collected across all three sub-cohorts of RS (RS-I, RS-II and RS-III). DNA was isolated from whole blood at a centralized laboratory at Erasmus MC using a manual salting-out protocol. Initially, DNA samples were stored in Eppendorf tubes at −20 °C, while in later study phases, storage transitioned to Matrix 2D-barcode tubes for improved tracking and management. The GWAS dataset comprises genotypes from over 12,000 DNA samples across the three RS cohorts. It includes both a pilot dataset and a large dataset of approximately 12,000 samples, genotyped using Illumina arrays: RS-I and RS-II: 550 K SNPs (single + duo array format); RS-III: 610 K SNPs (Quattro array format). The Illumina GWAS genotype data serve as the foundation for imputation analysis, enhancing genetic resolution. In this study, the RS GWAS datasets were imputed using the 1000 Genomes (1 KG) reference panel, version Iv3, resulting in a dataset of approximately 30 million SNP genotypes.^28^^,^^29^ A total of 241 conditionally independent genome-wide significant associations for coronary artery disease (CAD) were retrieved from the latest and largest GWAS in participants of predominantly European ancestry, with *p*-value threshold of 5 × 10^−8^.^15^^,^^30^ The reported linkage disequilibrium (LD) correlation coefficients (*r*) for these SNPs ranged from −0.36 to 0.21, corresponding to *r*^*2*^ values between 0.04 and 0.13, confirming low LD and the independence of the included variants. Of these, 225 Independent Significant SNPs (IndSigSNP) were available in the Rotterdam Study GWAS dataset, representing conditionally independent association signals identified during GWAS processing. These 225 SNPs were used to construct the polygenic risk score for coronary artery disease (PRS-CAD). For each participant, the PRS-CAD was calculated by multiplying the dosage of each risk allele by its corresponding effect estimate (weight) and summing the weighted alleles across all SNPs (Supplemental Excel).^13^ High genetic risk was defined based on the quintiles of PRS-CAD, we categorized participants into three genetic risk groups: low (lowest quintile), intermediate (20th–80th percentile), and high (highest quintile).

### Ascertainment of CHD

The primary outcome in this study is CHD, encompassing non-fatal myocardial infarction (MI) and surgical or percutaneous myocardial revascularization.^31^ Incident CHD was identified in all three cohorts through automated linkage of the study database to medical reports from general practitioners, nursing home physicians and hospitals, and annual collection of their medical records. All data on potential CHD events were independently adjudicated by two research physicians and subsequently verified by an experienced cardiologist. For each participant follow-up data were censored at the time of a first CHD event, last health status update, or at the end of the follow-up period (January 1, 2020), whichever occurred first. Information on prevalent CHD was ascertained during baseline interviews and confirmed by reviewing medical records.^31^

### Assessment of other baseline characteristics

Height and weight of the participants were measured during physical examinations at the research centre while wearing indoor clothes without shoes. Body mass index (BMI) was calculated as weight divided by height squared (kg/m^2^).^32^ Blood pressure was measured twice at the right brachial artery using a random-zero sphygmomanometer after participants had been seated quietly for at least 5 min and the mean of both readings was calculated.^33^ Fasting blood samples were collected and analysed using the glucose hexokinase method.^34^ Circulating cholesterol levels were assessed via an automated enzymatic method.^33^^,^^35^ Information on age, sex, educational level, whether participants used any food supplement was collected through questionnaires during home interviews. Current medication use was documented during these interviews and classified in accord with the Anatomical Therapeutic Chemical classification system. Hypertension at baseline was defined as a resting systolic blood pressure (SBP) ≥140 mmHg, diastolic blood pressure (DBP) ≥90 mmHg, and/or the use of blood pressure-lowering medication. Diabetes was defined as fasting glucose >6.9 mmol/L, non-fasting glucose >11.0 mmol/L, and/or the use of antidiabetic medication or insulin.

### Data processing and statistical analysis

Baseline characteristics of the study population were summarized using frequencies and percentages, means with standard deviations (SD), or medians with interquartile ranges (IQR). Cox proportional hazard regression model with age as underlying time variable was used to estimate adjusted hazard ratios (HRs) and 95% confidence intervals (CIs) of incident CHD for hPDI-Lifestyle score and PRS-CAD (categories and per standard deviation increment). The proportional hazards assumption was assessed using Grambsch-Therneau (G-T) test,^36^ and no evidence of violation was detected (data not shown). Three models were applied to Cox regression for association between hPDI-Lifestyle and risk of incident CHD: Model 1 is adjusted for age (years old), sex (men and women), sub-cohort (RS-I-1, RS-II-1 and RS-III-1), energy intake (kcal/day), margarine intake (gram/day) and miscellaneous foods intake (gram/day); Model 2 (main model) is additionally adjusted for education level (primary, secondary general or vocational education, higher vocational education or university), alcoholic beverage intake (0, 1≤ and >1 glass/day), any food supplement use (yes or no), serum lipid reducing agents use (yes or no), having diabetes (yes or no) and hypertension (yes or no); Model 3 (mediation) is further adjusted for BMI (kg/m^2^), which was considered a potential mediator to assess whether the association between hPDI-Lifestyle and CHD risk was independent of, or partly mediated through, BMI. For the analyses of genetic risk (PRS-CAD score) and risk of incident CHD, Cox regression models were adjusted for age and sex only. The Rotterdam Study included participants of predominantly European ancestry and was drawn from a geographically and ethnically homogeneous region, population stratification was expected to be minimal. Accordingly, adjustment for genetic principal components was not required in the current analyses.

We examined interaction of sex with hPDI-Lifestyle on CHD risk by including a multiplicative interaction term (hPDI-Lifestyle score × sex) in Model 2. Because there was no strong evidence to suggest a significant interaction between hPDI-Lifestyle score with sex for CHD (*p* for interaction = 0.43, Supplemental Figure S1), we did not stratify by sex and proceeded with analyses including both men and women together. To evaluate whether genetic predisposition to CHD modified the associations between the hPDI-Lifestyle score and CHD, we tested multiplicative interactions by adding product terms between the hPDI-Lifestyle score and the PRS (categories and continuous) in Model 2. Because of statistically significant interaction terms for hPDI-Lifestyle with genetic risk and to further evaluate these interactions, we subsequently conducted stratified analyses across genetic risk categories (high, intermediate, and low genetic risk groups based on PRS quintiles), with poor hPDI-Lifestyle at the high genetic risk and serving as the reference category to evaluate the joint effects genetic risk and hPDI-lifestyle on CHD risk.

We further evaluated the potential non-linear association between hPDI-Lifestyle score and PRS with CHD using restricted cubic spline regression. Multiple imputation was implemented to account for missing data on covariates (ranging from 0.3 to 35.5%, Supplemental Table S3; n = 30 imputations). To visualize the shape of associations, restricted cubic splines were fitted using one randomly selected imputed dataset. All reported hazard ratios and 95% confidence intervals were derived from pooled results across 30 imputed datasets following Rubin's rules.

### Sensitivity analyses

We performed several sets of sensitivity analyses based on Model 2. First, we repeated main analyses using complete case analyses. Second, we repeated the spline-curve modelling for the association between hPDI-Lifestyle and incident CHD using three additional randomly selected imputed datasets (n = 9, 17, and 28). Third, we excluded events occurring within the first 2 years of follow-up to reduce the influence of reverse causality. Forth, given the limited proportion of participants with data on smoking cessation duration as specified in the LE 8 metrics, we conducted additional analyses among those with available data (n = 3399 for CHD) to explore whether alternative categorizations of smoking status would yield different results. Smoking status was classified into five groups: never smoker, former smoker who quit ≥5 years ago, former smoker who quit 1 to <5 years ago, former smoker who quit <1 year ago, and current smoker. Last, we examined the combined effects of recommended dietary patterns, including DASH and Mediterranean Diet, in conjunction with other health behaviours, on CHD events.

Statistical analyses were performed with R version 3.1.2 (R Foundation for Statistical Computing, Vienna, Austria) and STATA SE 18.0 (Stata Corp). A two-sided *p* value < 0.05 was considered statistically significant.

### Role of the funding source

The sponsors had no role in the design and conduct of the study, collection, management, analysis and interpretation of the data, or preparation, review or approval of the manuscript.

## Results

### Population for analysis

**Fig. 1:**
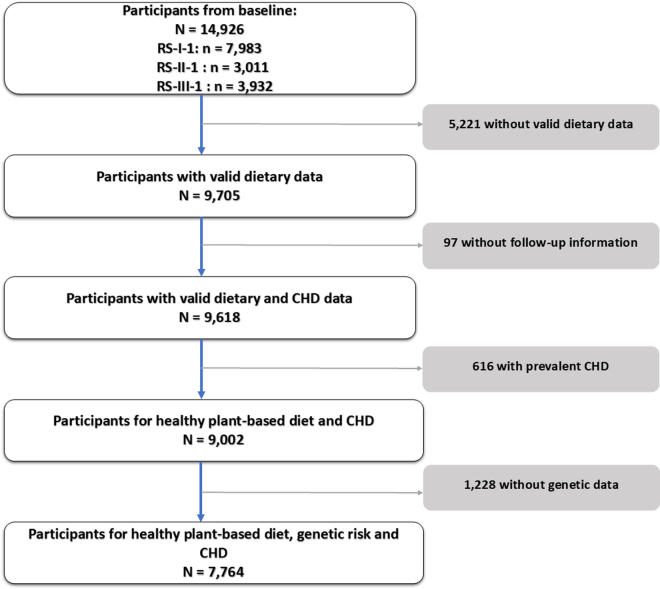
**Flowchart showing selection of participants from the Rotterdam Study included in the main analysis**.

Out of 14,926 participants, we had reliable dietary data available for 9705 participants (estimated energy intake >500 and < 5000 kcal/day) at baseline. Then, we excluded 97 participants without informed consent or valid follow-up data on CHD incidence, 616 with prevalent CHD at baseline, and 1228 without genetic data. This resulted in 7764 participants included in the analyses of the hPDI-Lifestyle score and incident CHD (Fig. 1).

### Participants’ characteristics

Baseline characteristics of participants are shown in Table 1. The majority of the cohort were women (4672 [60.1%]), under 65 years old (4679 [60.2%]), with secondary or vocational education (5364 [69.4%]), and non-drinkers (4749 [68.2%]). More than half of the participants had a higher BMI (3696 [47.8%] overweight and 1380 [17.8%] obese) and hypertension (4309 [55.7%]). The mean hPDI score was 66.7 (SD = 7.4). The median physical activity, measured in MET hours, was 69.5 (IQR: 39.5–102.3), while the median sleep duration was 7.0 h (IQR: 6.0–8.0). Approximately 45% of participants (N = 3483) were former smokers and 21.8% (N = 1686) were current smokers. In terms of hPDI-Lifestyle adherence, 14.0% (N = 1082) demonstrated poor adherence (scores 0–49), 65.2% (N = 5047) had intermediate adherence (scores 50–79), and 20.8% (N = 1609) achieved ideal adherence (scores 80–100).

**Table 1:** Baseline characteristics of 7764 participants from the Rotterdam study.

Characteristic	Overall	Adherence to hPDI-Lifestyle		
		Poor (0–49)	Intermediate (50–79)	Ideal (80–100)
Women, N (%)	4672 (60.1)	170 (43.4)	2928 (58.0)	1250 (77.7)
Age, years old, N (%)				
<65	4679 (60.2)	633 (58.5)	3025 (59.9)	1004 (62.4)
≥65	3095 (39.8)	449 (41.5)	2022 (40.1)	605 (37.6)
Education level, N (%)				
Primary	1179 (15.2)	178 (16.5)	766 (15.2)	232 (14.5)
Secondary general or vocational	5364 (69.4)	775 (71.7)	3480 (69.1)	1101 (68.7)
Higher vocational or university	1188 (15.4)	128 (11.8)	790 (15.7)	270 (16.8)
Alcohol intake, glass/day, N (%)				
0	4749 (68.2)	556 (55.8)	3072 (68.1)	1109 (77.7)
≤1	885 (12.7)	167 (16.7)	568 (12.6)	140 (9.8)
>1	1330 (19.1)	274 (27.5)	868 (19.3)	179 (12.5)
BMI, kg/m^2^, N (%)				
Underweight (≤18.5)	56 (0.7)	15 (1.4)	33 (0.7)	7 (0.4)
Healthy (>18.5–24.9)	2604 (33.7)	415 (38.6)	1621 (32.3)	550 (34.4)
Overweight (25–29.9)	3696 (47.8)	515 (47.9)	2443 (48.6)	724 (45.2)
Obese (≥30)	1380 (17.8)	130 (12.1)	927 (18.4)	320 (20.0)
Energy intake, kcal/day, median (IQR)	2024 (1687–2421)	2191 (1838–2599)	2042 (1712–2431)	1841 (1536–2224)
Any vitamin or mineral supplement use, yes, N (%)	1176 (15.2)	126 (11.7)	757 (15.0)	288 (18.0)
Diabetes, yes, N (%)	686 (10.9)	101 (13.6)	443 (10.7)	140 (10.0)
Hypertension, yes, N (%)	4309 (55.7)	574 (53.4)	2828 (56.3)	897 (55.7)
Serum lipid reducing agents use, yes, N (%)	622 (8.0)	49 (4.6)	414 (8.2)	159 (9.9)
Health Behaviours Metrics				
Adherence to hPDI, score, mean (SD)	66.7 (7.4)	61.4 (5.5)	65.8 (6.8)	73.1 (5.8)
Smoking status, N (%)				
Never	2569 (33.2)	4 (0.4)	1356 (26.9)	1209 (75.1)
Former	3483 (45.0)	349 (32.3)	2734 (54.2)	400 (24.9)
Current	1686 (21.8)	729 (67.4)	957 (19.0)	–
Physical activity, MET hours/week, median (IQR)	69 (39–102)	64 (33–92)	68 (39–101)	75 (47–110)
Sleep duration, hours/night, median (IQR)	7 (6–8)	5 (5–6)	7 (6–8)	8 (7–8)
hPDI-Lifestyle score, points, mean (SD)	65.8 (14.1)	43.3 (5.1)	64.4 (7.5)	85.4 (5.6)
Adherence to hPDI-Lifestyle, N (%)				
Poor (0–49)	1082 (14.0)	–	–	–
Intermediate (50–79)	5047 (65.2)	–	–	–
Ideal (80–100)	1609 (20.8)	–	–	–

Values are on the basis of unimputed data.

Abbreviations: BMI, body mass index; hPDI, healthful Plant-based Dietary Index; IQR, interquartile range; SD, standard deviation.

hPDI-lifestyle score incorporating four health behaviour components from Life's Essential 8 metrics: adherence to a healthy diet, physical activity, smoking status and sleep heath.

### hPDI-lifestyle and incident CHD

**Fig. 2:**
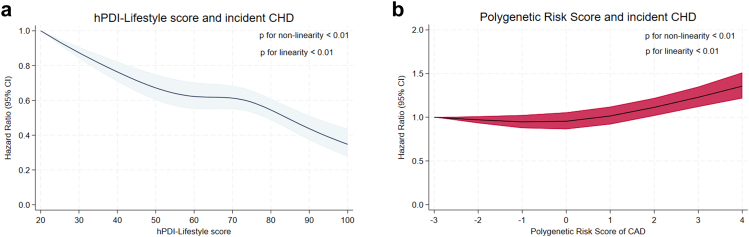
**Associations of healthy plant-based diet index-lifestyle score and polygenetic risk score with the risk of incident CHD in the Rotterdam Study**. a. hPDI-Lifestyle Score and Incident Coronary Heart Disease; b. Polygenetic Risk Score and Incident Coronary Heart Disease. The multivariable-adjusted HRs with 95% CIs of continuous healthy plant-based diet index-lifestyle scores are indicated by the navy blue line and light-blue shading respectively; HRs with 95% CIs were adjusted for sex, age (years old), sub-cohort (RS-I-1, RS-II-1 or RS-III-1), daily dietary energy intake (kcal/day), margarine intake (gram/day), miscellaneous foods intake (gram/day), education level (primary, secondary general or vocational education, higher vocational education or university), alcohol intake (0, ≤1, >1 glass/day), any vitamins supplement use (yes or no), diabetes (yes or no), hypertension (yes or no) and serum lipid reducing agents use (yes or no); The multivariable-adjusted HRs with 95% CIs of continuous polygenetic risk scores are indicated by the dark line and red shading respectively; HRs with 95% CIs were adjusted for sex and age (years old).

We documented 918 CHD cases, during a mean follow-up of 15.2 years (SD = 7.9), encompassing 116,324 person-years among 7764 participants. A higher hPDI-Lifestyle score was associated with a significantly lower risk of incident CHD (adjusted HR 0.91, 95% CIs 0.89–0.92 per SD increment in hPDI-Lifestyle score, Model 2; Table 2). Participants with scores reflecting ideal adherence to a hPDI-Lifestyle had a 22% lower risk of incident CHD compared to those with poor adherence (HR 0.78, 95% CI 0.73–0.83). We also confirmed that higher genetic risk for CHD, as assessed by PRS, was associated with an increased risk of incident CHD (HR 1.03, 95% CI 1.01–1.04 per SD increment in PRS, Table 2). Participants at high genetic risk had a 13% higher risk of incident CHD compared to those at low genetic risk (HR 1.13, 95% CI 1.07–1.18). These effect estimates remained consistent across different levels of adjustment for confounders (Model 1, Supplemental Table S5) and potential mediators (Model 3, Supplemental Table S6). Evidence of non-linearity was observed in the associations between hPDI-Lifestyle and incident CHD (*p* for non-linearity <0.01, Fig. 2a) as well as between PRS and incident CHD (*p* for non-linearity <0.01, Fig. 2b).

**Table 2:** Multivariable adjusted hazard ratios (HRs) with 95% confidence intervals (CIs) for incident CHD according to categorical and continuous hPDI-Lifestyle score and genetic risk in 7764 participants from the Rotterdam Study.

	HR (95% CI)
Incident cases	918
Incident rate, per 10,000 person-year	78.9
Adherence to hPDI-Lifestyle	
Poor (0–49)	Reference (1.00)
Intermediate (50–79)	0.87 (0.82, 0.92)
Ideal (80–100)	0.78 (0.73, 0.83)
Per SD increment in hPDI-Lifestyle score	0.91 (0.89, 0.92)
Genetic risk	
Low (Q1)	Reference (1.00)
Intermediate (Q2–Q4)	1.09 (1.05, 1.14)
High (Q5)	1.09 (1.04, 1.15)
Per SD increment in PRS	1.02 (1.00, 1.03)

Abbreviations: hPDI, healthful plant-based dietary index; PRS, polygenetic risk score.

For analysis on hPDI-Lifestyle and incident CHD, hazard ratios (HRs) with 95% confidence intervals (CIs) were adjusted for sex, age (years old), sub-cohort (RS-I-1, RS-II-1 or RS-III-1), daily dietary energy intake (kcal/day), margarine intake (gram/day), miscellaneous foods intake (gram/day), education level (primary, secondary general or vocational education, higher vocational education or university), alcohol intake (0, ≤1, >1 glass/day), any vitamins supplement use (yes or no), diabetes (yes or no), hypertension (yes or no) and serum lipid reducing agents use (yes or no).

For analysis on PRS-CAD and incident CHD, HRs with 95% CIs were adjusted for sex and age.

Effect estimates are based on pooled results of imputed data.

### Gene-lifestyle interactions and incident CHD

Table 3 demonstrates the association between adherence to the hPDI-Lifestyle and incident CHD, stratified by genetic risk. Compared with participants with a high genetic risk and poor adherence to the hPDI-Lifestyle, ideal adherence to the hPDI-Lifestyle was associated with a 20% lower CHD risk for those at low genetic risk (HR 0.80, 95% CI 0.71–0.90), a 20% lower CHD risk for those at intermediate genetic risk (HR 0.80, 95% CI 0.72–0.89) and a 44% lower CHD risk for those at high genetic risk (HR 0.56, 95% CI 0.49–0.64) (*p* for interaction between genetic risk and hPDI-Lifestyle <0.001; Supplemental Figure S2). Also, intermediate adherence to the hPDI-Lifestyle was associated with a lower risk of CHD risk among participants at low and intermediate genetic risk (HRs 0.75, 95% CIs 0.67–0.84 and 0.84, 0.76–0.93), while not for those at high genetic risk (HR 0.93, 95% CI 0.84–1.04). Poor adherence to hPDI-Lifestyle was associated with a lower risk of incident CHD risk only among participants at low genetic risk (HR 0.73, 95% CI 0.61–0.87). These findings were consistent across different levels of confounder adjustment (Model 1 and 3, Supplemental Tables S10 and S11).

**Table 3:** Multivariable adjusted hazard ratios (HRs) with 95% confidence intervals (CIs) for incident CHD according to categorical hPDI-Lifestyle score stratified by genetic risk in 7764 participants from the Rotterdam Study.

	HR (95% CI)
High genetic risk (Q5)	
Poor hPDI-Lifestyle (0–49)	Reference (1.00)
Intermediate hPDI-Lifestyle (50–79)	0.93 (0.84, 1.04)
Ideal hPDI-Lifestyle (80–100)	0.56 (0.49, 0.64)
Intermediate genetic risk (Q2–Q4)	
Poor hPDI-Lifestyle (0–49)	1.02 (0.90, 1.15)
Intermediate hPDI-Lifestyle (50–79)	0.84 (0.76, 0.93)
Ideal hPDI-Lifestyle (80–100)	0.80 (0.72, 0.89)
Low genetic risk (Q1)	
Poor hPDI-Lifestyle (0–49)	0.73 (0.61, 0.87)
Intermediate hPDI-Lifestyle (50–79)	0.75 (0.67, 0.84)
Ideal hPDI-Lifestyle (80–100)	0.80 (0.71, 0.90)

Abbreviation: hPDI, healthful plant-based dietary index.

All hazard ratios (HRs) with 95% confidence intervals (CIs) were adjusted for age (years old), sub-cohort (RS-I-1, RS-II-1 or RS-III-1), daily dietary energy intake (kcal/day), margarine intake (gram/day), miscellaneous foods intake (gram/day), education level (primary, secondary general or vocational education, higher vocational education or university), alcohol intake (0, ≤1, >1 glass/day), any vitamins supplement use (yes or no), diabetes (yes or no), hypertension (yes or no) and serum lipid reducing agents use (yes or no).

Effect estimates are based on pooled results of imputed data.

### Sensitivity analyses

Firstly, results were similar in complete-case analyses (Supplementary Tables S9 and S14). Secondly, nonlinear patterns remained consistent, supporting the stability of the visualized shapes of association across imputed data (Supplemental Figure S3). Thirdly, the associations between adherence to the hPDI-Lifestyle and incident CHD remained robust after excluding events occurring within the first two years of follow-up (Supplementary Tables S7 and S12). Fourthly, the results remained largely consistent when smoking status was classified into five groups though (Supplementary Tables S8 and S13). Similarly, we found that higher adherence to a DASH-Lifestyle and a Mediterranean Diet-Lifestyle was associated with a lower risk of CHD incidence (HR 0.92, 95% CI 0.91–0.94 per SD increment in DASH-Lifestyle score; HR 0.90, 95% CI 0.88–0.91 per SD increment in Mediterranean Diet-Lifestyle score, Model 2; Supplemental Table S15).

## Discussion

This population-based prospective cohort study, highlights the significance of adhering to a healthy lifestyle, as quantified by the hPDI-Lifestyle score, in reducing the risk of CHD. We observed that higher adherence to the hPDI-Lifestyle is associated with a lower risk of incident CHD over 30 years of follow-up, with the most pronounced reduced risk of CHD in individuals at high genetic risk. Our findings support recommendations to adopt a healthy plant-based diet, complemented by non-smoking, adequate physical activity and moderate sleep duration for CHD prevention. Furthermore, we provide novel insights into the interplay between lifestyle behaviours and genetic predisposition, suggesting that individuals with high genetic risk may derive the greatest benefit from adopting a healthy lifestyle in promoting cardiovascular health.

Our observed association between ideal hPDI-Lifestyle adherence and a decreased risk of incident CHD align with prior studies that also observe that healthier lifestyle may reduce CHD risk,^7^, ^8^, ^9^ even though the lifestyle behaviours included vary across these studies. Two studies conducted in the U.K and the U.S. defined health behaviours based on the American Heart Association's 2020 Strategic Impact Goal guidelines,^37^ incorporating diet, smoking, BMI, and physical activity.^7^^,^^8^ In contrast, a study conducted in China included five lifestyle factors, adding waist circumference.^9^ Furthermore, the included metric of diet also differs, with the two western studies defining a healthy diet as reaching recommended intake of at least half of the following food groups: increased consumption of fruits, nuts, vegetables, whole grains, fish, and dairy products and reduced or no consumption of refined grains, processed meats, unprocessed red meats, sugar-sweetened beverages.^7^^,^^8^ While the study conducted in China defined a healthy diet based on having any of three dietary habits: consuming fresh fruits daily, eating vegetables daily, or limiting red meat consumption to less than once weekly or not at all.^9^ In our study, we introduced the healthy plant-based diet index encompassing 22 commonly consumed food groups. This index enables a nuanced assessment of dietary patterns, highlighting more consumption of healthy plant-based foods, and less consumption of unhealthy plant-based and animal-based foods on a continuous scale. A body of evidence demonstrated that the healthful plant-based dietary pattern is associated with a lower risk of CHD and mortality,^10^^,^^21^ but no previous study integrated it with other non-dietary lifestyles. Our study, which integrate the hPDI with smoking status, and sleep health-into the hPDI-Lifestyle score, offers a more comprehensive evaluation of lifestyle behaviours and providing novel evidence on the link between hPDI-Lifestyle and incident CHD risk after adjusting for the other 4 health factors included in the Life's Essential 8. It is also important to acknowledge that comorbidities such as hypertension and diabetes may develop during follow-up and could partly mediate the association between lifestyle behaviours and CHD risk. Although we adjusted for these conditions at baseline, they may arise as intermediate outcomes influenced by lifestyle factors, subsequently contributing to CHD development. Our analyses were designed to estimate the total effect of baseline lifestyle on CHD risk, reflecting its overall preventive potential. Future studies incorporating repeated measures of lifestyle and metabolic risk factors could help disentangle these temporal pathways and quantify the mediating role of such comorbidities. We also observed similar associations between greater adherence to the DASH-Lifestyle and Mediterranean Diet-Lifestyle and a lower risk of CHD. Compared to these dietary patterns, the healthful plant-based dietary pattern provides a comprehensive evaluation of plant-based dietary quality by distinguishing between healthy and unhealthy plant-based foods. Such dietary patterns align with broader sustainability goals. Increasingly, science, policy, and industry sectors are working together to promote a transition toward sustainable, plant-forward diets that support both population and planetary health. For instance, national initiatives such as the Green Deal on Sustainable Healthcare in the Netherlands underscore the importance of integrating sustainability into dietary and healthcare practices, reflecting the growing recognition of the dual benefits of plant-based nutrition for human and environmental health.^38^

In addition to these main effects of lifestyle on incident CHD, we confirmed that a polygenetic risk score for CHD is positively associated with incident coronary events.^7^^,^^8^^,^^15^ The modest non-linearity observed in the PRS-CHD association may reflect a non-additive aggregation of risk alleles, where the combined effect of multiple genetic variants leads to a disproportionate increase in CHD risk at higher levels of polygenic burden. The overall association remained positive and consistent across PRS categories. We also observed a pronounced reduction in CHD risk with higher hPDI-Lifestyle adherence among individuals with high genetic predisposition. A few previous studies explored interactions between lifestyle and genetic risk, suggesting that while adherence to a healthy lifestyle reduce CHD risk, its effect is relatively consistent across genetic risk groups.^7^, ^8^, ^9^ However, these studies also indicated that individuals with high genetic risk experience greater benefits from lifestyle improvements in preventing CHD.^7^, ^8^, ^9^ Our study, constructing the PRS-CAD based on the latest GWAS findings, further refines this understanding by quantifying the interaction between genetic predisposition and healthy lifestyle behaviours, revealing that the potential protective effect of the ideal hPDI-Lifestyle adherence may be amplified in those at high genetic risk.

Beyond the interplay between genetic risk and behavioural lifestyle in relation to CHD events, genetic coronary artery calcium (CAC), quantified via computed tomography (CT), has been emerged as a robust marker of subclinical atherosclerosis. Compared to other risk markers (e.g., carotid intima-media thickness), CAC offers superior discrimination and classification for predicting clinical CHD risk.^28^^,^^39^ Whether an interaction exists between CAC burden and lifestyle factors in relation to CHD risk warrants further investigation. One prior study reported that a favourable lifestyle was associated with significantly lower levels of CAC within each genetic risk category, highlighting CAC not only as a potential effect modifier but also as a candidate mediator through which lifestyle factors may influence CHD risk.^8^ This suggests that the beneficial effects of healthy lifestyle adherence on overt CHD may be partially channelled through attenuation of subclinical atherosclerosis. Nevertheless, the mechanisms by which lifestyle influences CHD risk likely extend beyond structural coronary changes, encompassing systemic inflammation, lipid metabolism, and endothelial function.^40^ The cardioprotective effects of healthy lifestyle may also be explained by the nutrient composition of the healthy plant-based diet and associated biological pathways.^10^^,^^21^ Healthy plant-based diets rich in dietary fibre, antioxidants, and unsaturated fatty acids have been shown to beneficially modulate multiple mechanisms underlying coronary atherosclerosis.^40^ In addition, increased fibre intake promotes a diverse gut microbiome and enhances production of short-chain fatty acids, which can reduce systemic inflammation and improve lipid metabolism.^41^^,^^42^ Antioxidants and polyphenols abundant in healthy plant foods reduce oxidative stress and endothelial dysfunction,^43^^,^^44^ while unsaturated fatty acids improve lipid profiles and vascular health.^45^ Together, these mechanisms may mitigate genetic susceptibility by attenuating inflammation, improving endothelial function, and stabilizing atherosclerotic plaques. Future studies should further elucidate these underlying biological pathways.

One of the major strengths of this study is that, to our knowledge, it is the first to comprehensively assess lifestyle behaviours using the hPDI-Lifestyle score, based on a large population-based cohort study with a long follow-up period, integrating plant-based diet quality, physical activity, smoking status, and sleep duration. This holistic approach reflects the multifaceted nature of determinants of cardiovascular health. Furthermore, the stratification by genetic predisposition highlights the potential for tailoring lifestyle interventions based on individual genetic risk profile, supporting personalized recommendations for CHD prevention. Despite these strengths, several limitations warrant consideration. First, the observational design precludes causal inferences, and residual confounding cannot be ruled out despite extensive adjustments. Second, dietary intake and other lifestyle behaviours were self-reported, which may have introduced measurement bias. In addition, lifestyle behaviours were assessed only once at baseline, which may not fully capture potential changes over the long follow-up period. However, given that participants were older adults when they enrolled in this study, substantial alterations in their habitual behaviours are less likely. Third, although our study benefits from a large sample size, the lack of complete data on all relevant variables for all participants may have introduced selection bias, particularly in the analysis on coronary artery calcium with relevant smaller sample size. Nevertheless, a comparison of participant characteristics between those with complete and incomplete data revealed similar distributions of baseline variables. Fourth, although we possess extensive data for several variables in this study, it would have been ideal to account for detailed smoking cessation information, and this information was available for only a subset of the sample. Nevertheless, sensitivity analyses incorporating these data yielded similar results. Moreover, although we adjusted for baseline confounders including education level, metabolic disorder, medication use, potential residual confounding from unmeasured or incompletely measured factors such as disease progression or psychosocial stress cannot be entirely excluded. Lastly, our PRS was constructed using an LD clumping threshold of r^2^ < 0.01. While a more stringent cutoff (e.g., r^2^ < 0.001) is common in some PRS applications, our choice was informed by prior work of European-ancestry predominant populations and aimed at balancing variant independence with predictive information retention. Future studies in larger and more diverse cohorts may evaluate the impact of varying LD thresholds on predictive performance and explore the biological mechanisms underpinning the interactions between genetic predisposition and lifestyle factors. Additionally, investigating the role of other subclinical markers, such as inflammatory biomarkers and lipid metabolism by leveraging multi-omics data, could provide a better understanding of the pathways through which the hPDI-Lifestyle influences cardiovascular health.

In conclusion, our findings support recommendations to adopt a healthful plant-based diet in combination with non-smoking, adequate physical activity and moderate sleep duration for CHD prevention and indicate that this may be particularly relevant as prevention strategy for individuals with high genetic susceptibility to developing CHD. These results reinforce the importance of promoting holistic lifestyle interventions in cardiovascular disease prevention and support further research into tailoring lifestyle recommendations based on genetic predisposition.

## Contributors

XJW, TV and MS contributed to the study conception and design. Data collection was performed by TV, MK MG, MS, and DB, and analyses were conducted by XJW. The first draft of the manuscript was written by XJW and all authors commented on previous versions of the manuscript. XJW, TV, MK, MG, DB and MS verified the underlying data. NC provided support on revising manuscript. All authors read and approved the final manuscript. The corresponding authors (TV and MTS) had full access to all the data in the study and had final responsibility for the decision to submit for publication.

## Data sharing statement

The Rotterdam Study data are not publicly available due to privacy and ethical restrictions. Individual participant data that underlie the results reported in this article, after de-identification, may be made available to qualified researchers upon reasonable request, subject to a data sharing agreement and approval by the Rotterdam Study management team. Proposals should be directed to the corresponding authors of this article first. The study protocol and statistical analysis plan are available upon request.

## Declaration of generative AI and AI-assisted technologies in the writing process

During the preparation of this work the author(s) used ChatGPT in order to assist with drafting and refining text. After using this tool, the authors reviewed and edited the content as needed and take full responsibility for the content of the publication.

## Declaration of interests

The authors declare that they have no known competing financial interests or personal relationships that could have appeared to influence the work reported in this paper.

## Appendix A Supplementary data

Supplementary Figures and Tables

List of SNPs for PRS-CHD
