# Supplementary material for: Association between healthy plant-based diet-lifestyle (hPDI-Lifestyle) score and incidence of coronary heart disease, and effect modification by genetic predisposition: a prospective analysis in a population-based cohort
Source: Lancet Reg Health Eur. 2026 Feb 19;64:101619. doi: 10.1016/j.lanepe.2026.101619 (PMC12936782; doi:10.1016/j.lanepe.2026.101619)
Supplement: Supplementary Figures and Tables [file mmc1.docx]

**Supplemental Methods**

*Assessment of health behaviors and construction of a hPDI-Lifestyle score*

We constructed a hPDI-lifestyle score incorporating four health behavior components from Life’s Essential 8 (LE 8) metrics: adherence to a healthy diet, physical activity, smoking status and sleep health (Supplemental Table 2).^1^

Diet: LE8 supported the goal of pursuing DASH- and Mediterranean-style eating patterns for optimal cardiovascular health (CVH).^1^ Higher dietary pattern scores reflected greater intake of heart-healthy foods. A score of 100 points for the CVH diet metric should be assigned for those in the ≥95th percentile on the dietary pattern score (for populations). The 75th to 94th percentile should be assigned 80 points, given that improvement likely can be made even among those in this top quartile.^1^ In this study, the diet metric was based on hPDI, which was calculated by positively scoring intake of healthy plant-based food groups and reverse-scoring intake of unhealthy plant-based and animal-based groups across 22 food groups, summing to a total score of 22-110.^2^ The resulting hPDI was then categorized by study-population percentiles for analysis. Participants in the ≥95th percentile were assigned 100 points, those in the 75th-94th percentile received 80 points, 50 points were allocated to participants in the 50th-74th percentile, 25 points to those in the 25th-49th percentile, and 0 points to individuals in the 1st-24th percentile.

Physical activity: Thresholds were based in part on U.S. Physical Activity Guidelines.^1,3^ Each minute of moderate activity was counted as 1 minute, and each minute of vigorous activity as 2 minutes toward the weekly total. The scoring was non-linear to reflect diminishing marginal health benefits at higher activity levels.^1,3^ In this study, participants were scored according to their weekly accumulated moderate-intensity activity, measured in MET hours. Participants with ≥7·5 MET hours per week (equivalent to at least 150 minutes of activity) received 100 points, while those with 6-7·45, 4·5-5·95, 3-4·45, 1·5-2·95, and 0·05-1·45 MET hours per week were assigned 90, 80, 60, 40, and 20 points, respectively. Participants reporting no physical activity (0 MET hours) were given 0 points.

Nicotine exposure: Following LE8 recommendations, were subtracted for exposure to indoor secondhand smoke given its potential for long-term effects on cardiopulmonary health.^1,4^ In this study, we assigned never smokers 100 points, former smokers 50 points, and current smokers 0 points.

Sleep health: Scoring thresholds were based on established sleep guidelines, reflecting the inverse U-shaped association between sleep duration and health outcomes, such that excessive sleep duration is also considered to be suboptimal for CVH.⁵⁻⁷ Optimal CVH was associated with 7-8 hours of sleep per night.^5-7^ In this study, the scoring was based on average nightly sleep duration. Participants who sleep 7 to less than 9 hours per night received 100 points. Sleep durations of 9 to 10 hours scored 90 points, 6 to 7 hours 70 points, and 5 to 6 hours or 10 or more hours 40 points. Those sleeping 4 to 5 hours earned 20 points, while participants with less than 4 hours of sleep per night were assigned 0 points (Supplemental Table 2).

**References**

1. Lloyd-Jones DM, Allen NB, Anderson CAM, et al. Life's Essential 8: Updating and Enhancing the American Heart Association's Construct of Cardiovascular Health: A Presidential Advisory From the American Heart Association. *Circulation* 2022; **146**(5): e18-e43.

2. Wang Y, Liu B, Han H, et al. Associations between plant-based dietary patterns and risks of type 2 diabetes, cardiovascular disease, cancer, and mortality - a systematic review and meta-analysis. *Nutr J* 2023; **22**(1): 46.

3. Piercy KL, Troiano RP, Ballard RM, et al. The Physical Activity Guidelines for Americans. *Jama* 2018; **320**(19): 2020-8.

4. Raghuveer G, White DA, Hayman LL, et al. Cardiovascular Consequences of Childhood Secondhand Tobacco Smoke Exposure: Prevailing Evidence, Burden, and Racial and Socioeconomic Disparities: A Scientific Statement From the American Heart Association. *Circulation* 2016; **134**(16): e336-e59.

5. Hirshkowitz M, Whiton K, Albert SM, et al. National Sleep Foundation's updated sleep duration recommendations: final report. *Sleep Health* 2015; **1**(4): 233-43.

6. St-Onge MP, Grandner MA, Brown D, et al. Sleep Duration and Quality: Impact on Lifestyle Behaviors and Cardiometabolic Health: A Scientific Statement From the American Heart Association. *Circulation* 2016; **134**(18): e367-e86.

7. Watson NF, Badr MS, Belenky G, et al. Recommended Amount of Sleep for a Healthy Adult: A Joint Consensus Statement of the American Academy of Sleep Medicine and Sleep Research Society. *Sleep* 2015; **38**(6): 843-4.

**Supplemental Table 1.** Food items included in 22 PDI food groups and scoring methods of developing the healthy plant-based diet index.

| Food categories | Food groups | Key food items | Healthy plant-based diet index |
| --- | --- | --- | --- |
|  |  | |  |
|  | Whole grains | Whole grain, bread, dark bread, rye bread, whole grain breakfast oats, whole grain pasta, brown rice | **+** |
|  | Fruits | Apple, banana, pear, orange, strawberry, grapes, other fruits | **+** |
|  | Vegetables | Cauliflower, broccoli, spinach, carrots, onion, lettuce, tomato, cabbage, cooked vegetables | **+** |
| **Healthy plant-based food category** | Nuts | Peanuts, walnuts, other nuts, peanut butter | **+** |
|  | Legumes | Legumes, tofu, soybeans, other soy products | **+** |
|  | Vegetable oil | Olive oil and vegetable oils used for cooking | **+** |
|  | Tea and coffee | Black tea, green tea, herbal tea, coffee | **+** |
|  |  | |  |
|  | Refined grains | Cornflakes, white bread, croissants, raisin bread, white pasta, white rice | **-** |
| **Unhealthy plant-based food category** | Potatoes | Potatoes, fries | **-** |
|  | Sugary beverages | Carbonated beverages with sugar, non-carbonated beverages with sugar, orange juice, fruit juice | **-** |
|  | Sweets | Sugar, cookies, cake, chocolate, candy-bars, honey, sweets, chocolate toppings, other sweet toppings | **-** |
|  |  | |  |
|  | Low-fat milk | Skimmed milk, semi-skimmed milk, skimmed coffee creamer, semi-skimmed coffee creamer | **-** |
|  | Low-fat yoghurt | Skimmed yoghurt, semi-skimmed yoghurt, skimmed quark, buttermilk | **-** |
|  | Full-fat milk | Full-fat milk, cream, coffee-cream | **-** |
|  | Full-fat yoghurt | Full-fat yoghurt, semi-skimmed quark, full quark | **-** |
|  | Cheese | Full fat cheese, low fat cheese, cheese fondue, other cheese | **-** |
| **Animal-based food category** | Fish | Salmon, tuna, trout, herring, mussels, other fish | **-** |
|  | Eggs | Boiled eggs, fried eggs | **-** |
|  | Unprocessed white meat | Chicken | **-** |
|  | Animal fat | Butter on bread, butter used for cooking, lard | **-** |
|  | Processed and red meat | Beef, pork, meatballs, sate, bacon, liver, processed meats | **-** |
|  | Desserts and sugary dairy | Custard, cream, ice cream, mousse, cream, chocolate milk, fruit yoghurt, yoghurt drinks | **-** |

Healthy plant-based diet index, positive scores were assigned to the healthy plant-based food groups (Q1=1, Q5=5), and reverse scores were given to the unhealthy plant-based and animal-based food groups (Q1=5, Q5=1).

The range of three plant-based diet indices was from 22 to 110.

**Supplemental Table 2.** Health behavior components of Life’s Essential 8 Metrics for cardiovascular health.

| CVH metric | Method of measurement | Quantification of CVH metric: adults (≥20 y of age) | |
| --- | --- | --- | --- |
| Diet | Measurement: Self-reported dietary intake  Tool for measurement: semi-quantitative FFQ | Quantiles of hPDI score | |
|  |  | Scoring:  Points  100  80  50  25  0 | Quantile  ≥ 95^th^ percentile (top/ideal diet)  75^th^-94^th^ percentile  50^th^-74^th^ percentile  25^th^-49^th^ percentile  1^st^-24^th^ percentile (bottom/ least ideal quartile) |
| Physical activity | Measurement: Self-reported hours of PA per week  Tool for measurement: adapted version of the Zutphen Physical Activity Questionnaire and the LASA Physical Activity Questionnaire; and PAs were weighted according to intensity (MET) using Compendium of Physical Activities version 2011. | MET hours per week | |
|  |  | Scoring:  Points  100  90  80  60  40  20  0 | MET hours/week  ≥ 7·5  6-7·45  4·5-5·95  3-4·45  1·5-2·95  0·05-1·45  0 |
| Smoking status | Measurement: Self-reported use of cigarettes or inhaled  NDS  Tool for measurement: questionnaires during home interviews. Example questions: “Current cigarette smoker?”, “Past cigarette smoker?”, “How old were you when you quit smoking cigarettes?” | Scoring:  Points  100  [75  50  25]  50  0 | Status  Never smoker  Former smoker, quit ≥5 y  Former smoker, quit 1-<5 y  Former smoker, quit <1 y  Former smoker  Current smoker |
|  |  |  |  |
| Sleep health | Measurement: Self-reported average hours of sleep per night  Tool for measurement: questionnaires during home interviews. Example questions: “How long did you usually sleep per night (in hours)?” | Scoring:  Points  100  90  70  40  20  0 | Level  7-<9  9-<10  6-<7  5-<6 or ≥10  4-<5  <4 |

Abbreviations: CVH, cardiovascular health; hPDI, healthy plant-based diet index; food frequency questionnaire (FFQ); Metabolic Equivalent of Task (MET)

The overall hPDI-Lifestyle score of 80 to 100 be considered high CVH; 50 to 79, moderate CVH; and 0 to 49 points, low CVH.

**Supplemental Table 3a.** Numbers and percentages of participants with each variable with missing values and its imputation methods for analysis on hPDI-Lifestyle score and genetic risk with incident CHD when smoking status was 3 categories.

| Variables | NA (N) | NA (%) | Method for imputation |
| --- | --- | --- | --- |
| Food supplement use | 24 | 0·3 | logistic regression |
| Serum lipid reducing agents use | 24 | 0·3 | logistic regression |
| Smoking status | 36 | 0·5 | multinomial logistic regression |
| Alcohol intake | 810 | 10·4 | ordered logistic regression |
| Education level | 43 | 0·5 | ordered logistic regression |
| Diabetes | 1,468 | 18·9 | logistic regression |
| Hypertension | 36 | 0·5 | logistic regression |
| Body mass index | 38 | 0·5 | linear regression |
| Physical activity | 1,395 | 17·9 | predictive mean matching |
| Sleep | 2,760 | 35·5 | ordered logistic regression |

Abbreviations: NA, not available

Smoking status was categorized into 3 groups: never, former and current.

**Supplemental Table 3b.** Numbers and percentages of participants with each variable with missing values and its imputation methods for analysis on hPDI-Lifestyle score and genetic risk with incident CHD when smoking status was 5 categories.

| Variables | NA (N) | NA (%) | Method for imputation |
| --- | --- | --- | --- |
| Food supplement use | 21 | 0·6 | logistic regression |
| Serum lipid reducing agents use | 21 | 0·6 | logistic regression |
| Smoking status | 6 | 0·2 | ordered logistic regression |
| Alcohol intake | 810 | 23·8 | ordered logistic regression |
| Education level | 18 | 0·5 | ordered logistic regression |
| Hypertension | 20 | 0·6 | logistic regression |
| Body mass index | 12 | 0·3 | linear regression |
| Physical activity | 228 | 6·7 | predictive mean matching |
| Sleep | 139 | 4·1 | ordered logistic regression |

Abbreviations: NA, not available

Smoking status was categorized into 5 groups: never, former (quit ≥5 years), former (quit 1-<5 years), former (<1 years) and current.

**Supplemental Table 4.** Baseline Characteristics of 7,162 Excluded Participants from the Rotterdam Study.

| Characteristic |  |
| --- | --- |
| Women, % | 58·0 |
| Age, years old, % |  |
| <65 | 46·5 |
| ≥65 | 53·5 |
| Education level, % |  |
| Primary | 22·7 |
| Secondary general or vocational | 63·3 |
| Higher vocational or university | 14·0 |
| Alcohol intake, glass/day, % |  |
| 0 | 73·9 |
| ≤1 | 9·2 |
| >1 | 16·9 |
| BMI, kg/m^2^, % |  |
| Underweight (≤18·5) | 1·1 |
| Healthy (>18·5-24·9) | 31·9 |
| Overweight (25-29·9) | 46·2 |
| Obese (≥30) | 20·8 |
| Energy intake, kcal/day, median (IQR) | 2007 (1681-2370) |
| Any vitamin or mineral supplement use, yes, % | 16·0 |
| Diabetes, yes, % | 14·0 |
| Hypertension, yes, % | 68·0 |
| Serum lipid reducing agents use, yes, % | 11·4 |
| Health Behaviors Metrics |  |
| Adherence to hPDI, score, mean (SD) | 67·2 (7·6) |
| Smoking status, % |  |
| Never | 33·2 |
| Former | 45·2 |
| Current | 21·6 |
| Physical activity, MET hours/week, median (IQR) | 64·9 (36·2-96·6) |
| Sleep duration, hours/night, median (IQR) | 7·0 (6·0-8·0) |
| hPDI-Lifestyle score, points, mean (SD) | 74·9 (13·3) |
| Adherence to hPDI-Lifestyle, % |  |
| Poor (0-49) | 4·1 |
| Intermediate (50-79) | 52·0 |
| Ideal (80-100) | 43·9 |

Values are on the basis of unimputed data.

Abbreviations: BMI, body mass index; hPDI, healthful Plant-based Dietary Index; IQR, interquartile range; SD, standard deviation.

hPDI-lifestyle score incorporating four health behavior components from Life’s Essential 8 metrics: adherence to a healthy diet, physical activity, smoking status and sleep heath.

**Supplemental Table 5.** Hazard ratios (HRs) with 95% confidence intervals (CIs) for incident CHD according to categorical and continuous hPDI-Lifestyle score, adjusted for sex, age, sub-cohort, energy intake, margarine intake and miscellaneous foods intake (Model 1).

|  | HR (95% CI) |
| --- | --- |
| **Adherence to hPDI-Lifestyle** |  |
| Poor (0-49) | Reference (1·00) |
| Intermediate (50-79) | 0·87 (0·83, 0·92) |
| Ideal (80-100) | 0·78 (0·74, 0·83) |
| Per SD increment in hPDI-Lifestyle score | 0·91 (0·90, 0·93) |

Abbreviations: hPDI, healthful plant-based dietary index.

For analysis on hPDI-Lifestyle and incident CHD, hazard ratios (HRs) with 95% confidence intervals (CIs) were adjusted for sex, age (years old), sub-cohort (RS-I-1, RS-II-1 or RS-III-1), daily dietary energy intake (kcal/day), margarine intake (gram/day) and miscellaneous foods intake (gram/day);

Effect estimates are based on pooled results of imputed data.

**Supplemental Table 6.** Hazard ratios (HRs) with 95% confidence intervals (CIs) for incident CHD according to categorical and continuous hPDI-Lifestyle score, additionally adjusted for body mass index (Model 3).

|  | HR (95% CI) |
| --- | --- |
| **Adherence to hPDI-Lifestyle** |  |
| Poor (0-49) | Reference (1·00) |
| Intermediate (50-79) | 0·88 (0·83, 0·93) |
| Ideal (80-100) | 0·79 (0·74, 0·84) |
| Per SD increment in hPDI-Lifestyle score | 0·91 (0·89, 0·93) |

Abbreviations: hPDI, healthful plant-based dietary index.

For analysis on hPDI-Lifestyle and incident CHD, hazard ratios (HRs) with 95% confidence intervals (CIs) were adjusted for sex, age (years old), sub-cohort (RS-I-1, RS-II-1 or RS-III-1), daily dietary energy intake (kcal/day), margarine intake (gram/day), miscellaneous foods intake (gram/day), education level (primary, secondary general or vocational education, higher vocational education or university), alcohol intake (0, ≤1, >1 glass/day), any vitamins supplement use (yes or no), diabetes (yes or no), hypertension (yes or no), serum lipid reducing agents use (yes or no) and body mass index (kg/m^2^);

Effect estimates are based on pooled results of imputed data.

**Supplemental Table 7.** Multivariable adjusted hazard ratios (HRs) with 95% confidence intervals (CIs) for incident CHD according to categorical and continuous hPDI-Lifestyle score and genetic risk in 7,582 participants from the Rotterdam Study after excluding first 2 years of occurred cases.

|  | HR (95% CI) |
| --- | --- |
| Incident cases | 835 |
| Incident rate, per 10,000 person-year | 71·9 |
| **Adherence to hPDI-Lifestyle** |  |
| Poor (0-49) | Reference (1·00) |
| Intermediate (50-79) | 0·88 (0·83, 0·94) |
| Ideal (80-100) | 0·73 (0·68, 0·78) |
| Per SD increment in hPDI-Lifestyle score | 0·88 (0·87, 0·90) |
| **Genetic risk** |  |
| Low (Q1) | Reference (1·00) |
| Intermediate (Q2-Q4) | 1·11 (1·06, 1·16) |
| High (Q5) | 1·12 (1·06, 1·17) |
| Per SD increment in polygenetic risk score | 1·01 (1·00, 1·03) |

Abbreviations: hPDI, healthful plant-based dietary index.

For analysis on hPDI-Lifestyle and incident CHD, hazard ratios (HRs) with 95% confidence intervals (CIs) were adjusted for sex, age (years old), sub-cohort (RS-I-1, RS-II-1 or RS-III-1), daily dietary energy intake (kcal/day), margarine intake (gram/day), miscellaneous foods intake (gram/day), education level (primary, secondary general or vocational education, higher vocational education or university), alcohol intake (0, ≤1, >1 glass/day), any vitamins supplement use (yes or no), diabetes (yes or no), hypertension (yes or no) and serum lipid reducing agents use (yes or no);

For analysis on PRS-CAD and incident CHD, HRs with 95% CIs were adjusted for sex and age.

Effect estimates are based on pooled results of imputed data.

**Supplemental Table 8.** Multivariable adjusted hazard ratios (HRs) with 95% confidence intervals (CIs) for incident CHD according to categorical and continuous hPDI-Lifestyle score and genetic risk in 3,399 participants from the Rotterdam Study when smoking status was 5 categories.

|  | HR (95% CI) |
| --- | --- |
| Incident cases | 274 |
| Incident rate, per 10,000 person-year | 62·2 |
| **Adherence to hPDI-Lifestyle** |  |
| Poor (0-49) | Reference (1·00) |
| Intermediate (50-79) | 0·70 (0·61, 0·79) |
| Ideal (80-100) | 0·69 (0·61, 0·79) |
| Per SD increment in hPDI-Lifestyle score | 0·89 (0·86, 0·93) |
| **Genetic risk** |  |
| Low (Q1) | Reference (1·00) |
| Intermediate (Q2-Q4) | 1·23 (1·13, 1·34) |
| High (Q5) | 1·07 (0·96, 1·18) |
| Per SD increment in polygenetic risk score | 1·04 (1·01, 1·08) |

Abbreviations: hPDI, healthful plant-based dietary index.

For analysis on hPDI-Lifestyle and incident CHD, hazard ratios (HRs) with 95% confidence intervals (CIs) were adjusted for sex, age (years old), sub-cohort (RS-I-1, RS-II-1 or RS-III-1), daily dietary energy intake (kcal/day), margarine intake (gram/day), miscellaneous foods intake (gram/day), education level (primary, secondary general or vocational education, higher vocational education or university), alcohol intake (0, ≤1, >1 glass/day), any vitamins supplement use (yes or no), diabetes (yes or no), hypertension (yes or no) and serum lipid reducing agents use (yes or no);

For analysis on PRS-CAD and incident CHD, HRs with 95% CIs were adjusted for sex and age.

Effect estimates are based on pooled results of imputed data.

**Supplemental Table 9.** Multivariable adjusted hazard ratios (HRs) with 95% confidence intervals (CIs) for incident CHD according to categorical hPDI-Lifestyle score and genetic risk in 5,423 participants from the Rotterdam Study in complete case analysis.

|  | HR (95% CI) |
| --- | --- |
| Incident cases | 648 |
| Incident rate, per 10,000 person-year | 75·5 |
| **Adherence to hPDI-Lifestyle** |  |
| Poor (0-49) | Reference (1·00) |
| Intermediate (50-79) | 0·86 (0·69, 1·07) |
| Ideal (80-100) | 0·76 (0·57, 1·01) |
| Per SD increment in hPDI-Lifestyle score | 0·90 (0·83, 0·98) |
| **Genetic risk** |  |
| Low (Q1) | Reference (1·00) |
| Intermediate (Q2-Q4) | 1·08 (0·89, 1·32) |
| High (Q5) | 1·12 (0·89, 1·41) |
| Per SD increment in polygenetic risk score | 1·03 (0·96, 1·11) |

Abbreviations: hPDI, healthful plant-based dietary index.

For analysis on hPDI-Lifestyle and incident CHD, hazard ratios (HRs) with 95% confidence intervals (CIs) were adjusted for sex, age (years old), sub-cohort (RS-I-1, RS-II-1 or RS-III-1), daily dietary energy intake (kcal/day), margarine intake (gram/day), miscellaneous foods intake (gram/day), education level (primary, secondary general or vocational education, higher vocational education or university), alcohol intake (0, ≤1, >1 glass/day), any vitamins supplement use (yes or no), diabetes (yes or no), hypertension (yes or no) and serum lipid reducing agents use (yes or no);

For analysis on PRS and incident CHD, HRs with 95% CIs were adjusted for sex and age.

**Supplemental Table 10.** Multivariable adjusted hazard ratios (HRs) with 95% confidence intervals (CIs) for incident CHD according to categorical hPDI-Lifestyle score stratified by genetic risk, adjusted for sex, age, sub-cohort, energy intake, margarine intake and miscellaneous foods intake (Model 1).

|  | HR (95% CI) |
| --- | --- |
| **High genetic risk (Q5)** |  |
| Poor hPDI-lifestyle (0-49) | Reference (1·00) |
| Intermediate hPDI-lifestyle (50-79) | 0·92 (0·83, 1·02) |
| Ideal hPDI-lifestyle (80-100) | 0·54 (0·48, 0·62) |
| **Intermediate genetic risk (Q2-Q4)** |  |
| Poor hPDI-lifestyle (0-49) | 1·01 (0·90, 1·14) |
| Intermediate hPDI-lifestyle (50-79) | 0·84 (0·76, 0·93) |
| Ideal hPDI-lifestyle (80-100) | 0·80 (0·72, 0·89) |
| **Low genetic risk (Q1)** |  |
| Poor hPDI-lifestyle (0-49) | 0·73 (0·61, 0·86) |
| Intermediate hPDI-lifestyle (50-79) | 0·75 (0·68, 0·84) |
| Ideal hPDI-lifestyle (80-100) | 0·81 (0·72, 0·91) |

Abbreviations: hPDI, healthful plant-based dietary index.

Effect estimates are based on pooled results of imputed data.

**Supplemental Table 11.** Multivariable adjusted hazard ratios (HRs) with 95% confidence intervals (CIs) for incident CHD according to categorical hPDI-Lifestyle score stratified by genetic risk, additionally adjusted for body mass index (Model 3).

|  | HR (95% CI) |
| --- | --- |
| **High genetic risk (Q5)** |  |
| Poor hPDI-lifestyle (0-49) | Reference (1·00) |
| Intermediate hPDI-lifestyle (50-79) | 0·89 (0·80, 0·99) |
| Ideal hPDI-lifestyle (80-100) | 0·47 (0·41, 0·53) |
| **Intermediate genetic risk (Q2-Q4)** |  |
| Poor hPDI-lifestyle (0-49) | 0·97 (0·86, 1·09) |
| Intermediate hPDI-lifestyle (50-79) | 0·78 (0·70, 0·86) |
| Ideal hPDI-lifestyle (80-100) | 0·66 (0·59, 0·73) |
| **Low genetic risk (Q1)** |  |
| Poor hPDI-lifestyle (0-49) | 0·69 (0·58, 0·82) |
| Intermediate hPDI-lifestyle (50-79) | 0·71 (0·64, 0·79) |
| Ideal hPDI-lifestyle (80-100) | 0·65 (0·58, 0·74) |

Abbreviations: hPDI, healthful plant-based dietary index.

All hazard ratios (HRs) with 95% confidence intervals (CIs) were adjusted for age (years old), sub-cohort (RS-I-1, RS-II-1 or RS-III-1), daily dietary energy intake (kcal/day), margarine intake (gram/day), miscellaneous foods intake (gram/day), education level (primary, secondary general or vocational education, higher vocational education or university), alcohol intake (0, ≤1, >1 glass/day), any vitamins supplement use (yes or no), diabetes (yes or no), hypertension (yes or no), serum lipid reducing agents use (yes or no) and body mass index (kg/m^2^).

Effect estimates are based on pooled results of imputed data.

**Supplemental Table 12.** Multivariable adjusted hazard ratios (HRs) with 95% confidence intervals (CIs) for incident CHD according to categorical hPDI-Lifestyle score stratified by genetic risk in 7,582 participants from the Rotterdam Study, after excluding first 2 years of occurred cases.

|  | HR (95% CI) |
| --- | --- |
| **High genetic risk (Q5)** |  |
| Poor hPDI-lifestyle (0-49) | Reference (1·00) |
| Intermediate hPDI-lifestyle (50-79) | 0·89 (0·79, 0·99) |
| Ideal hPDI-lifestyle (80-100) | 0·43 (0·37, 0·49) |
| **Intermediate genetic risk (Q2-Q4)** |  |
| Poor hPDI-lifestyle (0-49) | 0·89 (0·79, 1·01) |
| Intermediate hPDI-lifestyle (50-79) | 0·76 (0·68, 0·85) |
| Ideal hPDI-lifestyle (80-100) | 0·69 (0·61, 0·77) |
| **Low genetic risk (Q1)** |  |
| Poor hPDI-lifestyle (0-49) | 0·64 (0·53, 0·77) |
| Intermediate hPDI-lifestyle (50-79) | 0·68 (0·61, 0·77) |
| Ideal hPDI-lifestyle (80-100) | 0·68 (0·60, 0·77) |

Abbreviations: hPDI, healthful plant-based dietary index.

All hazard ratios (HRs) with 95% confidence intervals (CIs) were adjusted for age (years old), sub-cohort (RS-I-1, RS-II-1 or RS-III-1), daily dietary energy intake (kcal/day), margarine intake (gram/day), miscellaneous foods intake (gram/day), education level (primary, secondary general or vocational education, higher vocational education or university), alcohol intake (0, ≤1, >1 glass/day), any vitamins supplement use (yes or no), diabetes (yes or no), hypertension (yes or no) and serum lipid reducing agents use (yes or no).

Effect estimates are based on pooled results of imputed data.

**Supplemental Table 13.** Multivariable adjusted hazard ratios (HRs) with 95% confidence intervals (CIs) for incident CHD according to categorical hPDI-Lifestyle score stratified by genetic risk in 3,399 participants from the Rotterdam Study when smoking status was 5 categories.

|  | HR (95% CI) |
| --- | --- |
| **High genetic risk (Q5)** |  |
| Poor hPDI-lifestyle (0-49) | Reference (1·00) |
| Intermediate hPDI-lifestyle (50-79) | 0·74 (0·58, 0·95) |
| Ideal hPDI-lifestyle (80-100) | 0·43 (0·33, 0·57) |
| **Intermediate genetic risk (Q2-Q4)** |  |
| Poor hPDI-lifestyle (0-49) | 1·02 (0·77, 1·35) |
| Intermediate hPDI-lifestyle (50-79) | 0·76 (0·60, 0·96) |
| Ideal hPDI-lifestyle (80-100) | 0·76 (0·60, 0·97) |
| **Low genetic risk (Q1)** |  |
| Poor hPDI-lifestyle (0-49) | 0·96 (0·65, 1·41) |
| Intermediate hPDI-lifestyle (50-79) | 0·50 (0·39, 0·65) |
| Ideal hPDI-lifestyle (80-100) | 0·86 (0·66, 1·11) |

Abbreviations: hPDI, healthful plant-based dietary index.

All hazard ratios (HRs) with 95% confidence intervals (CIs) were adjusted for age (years old), sub-cohort (RS-I-1, RS-II-1 or RS-III-1), daily dietary energy intake (kcal/day), margarine intake (gram/day), miscellaneous foods intake (gram/day), education level (primary, secondary general or vocational education, higher vocational education or university), alcohol intake (0, ≤1, >1 glass/day), any vitamins supplement use (yes or no), diabetes (yes or no), hypertension (yes or no) and serum lipid reducing agents use (yes or no).

Effect estimates are based on pooled results of imputed data.

**Supplemental Table 14.** Multivariable adjusted hazard ratios (HRs) with 95% confidence intervals (CIs) for incident CHD according to categorical hPDI-Lifestyle score stratified by genetic risk in 5,423 participants from the Rotterdam Study in complete case analysis.

|  | HR (95% CI) |
| --- | --- |
| **High genetic risk (Q5)** |  |
| Poor hPDI-lifestyle (0-49) | Reference (1·00) |
| Intermediate hPDI-lifestyle (50-79) | 0·71 (0·46, 1·08) |
| Ideal hPDI-lifestyle (80-100) | 0·62 (0·35, 1·10) |
| **Intermediate genetic risk (Q2-Q4)** |  |
| Poor hPDI-lifestyle (0-49) | 0·72 (0·45, 1·13) |
| Intermediate hPDI-lifestyle (50-79) | 0·67 (0·45, 0·998) |
| Ideal hPDI-lifestyle (80-100) | 0·60 (0·38, 0·95) |
| **Low genetic risk (Q1)** |  |
| Poor hPDI-lifestyle (0-49) | 0·78 (0·43, 1·40) |
| Intermediate hPDI-lifestyle (50-79) | 0·66 (0·43, 1·03) |
| Ideal hPDI-lifestyle (80-100) | 0·56 (0·32, 1·00) |

Abbreviations: hPDI, healthful plant-based dietary index.

All hazard ratios (HRs) with 95% confidence intervals (CIs) were adjusted for age (years old), sub-cohort (RS-I-1, RS-II-1 or RS-III-1), daily dietary energy intake (kcal/day), margarine intake (gram/day), miscellaneous foods intake (gram/day), education level (primary, secondary general or vocational education, higher vocational education or university), alcohol intake (0, ≤1, >1 glass/day), any vitamins supplement use (yes or no), diabetes (yes or no), hypertension (yes or no) and serum lipid reducing agents use (yes or no).

Effect estimates are based on pooled results of imputed data.

**Supplemental Table 15.** Multivariable adjusted hazard ratios (HRs) with 95% confidence intervals (CIs) for incident CHD according to categorical and continuous Dietary Approaches to Stop Hypertension and Mediterranean Diet score in 7,764 participants from the Rotterdam Study.

|  | HR (95% CI) |  |
| --- | --- | --- |
| **Adherence to** **DASH-Lifestyle** |  |  |
| Poor (0-49) | Reference (1·00) |  |
| Intermediate (50-79) | 0·75 (0·72, 0·79) |  |
| Ideal (80-100) | 0·76 (0·72, 0·81) |  |
| Per SD increment in DASH-Lifestyle score | | 0·92 (0·91, 0·94) |
| **Adherence to Mediterranean Diet-Lifestyle** |  |  |
| Poor (0-49) | Reference (1·00) |  |
| Intermediate (50-79) | 0·84 (0·79, 0·88) |  |
| Ideal (80-100) | 0·72 (0·68, 0·77) |  |
| Per SD increment in Mediterranean Diet-Lifestyle | | 0·90 (0·88, 0·91) |

Abbreviations: DASH, Dietary Approaches to Stop Hypertension.

For analysis on diet score and incident CHD, hazard ratios (HRs) with 95% confidence intervals (CIs) were adjusted for sex, age (years old), sub-cohort (RS-I-1, RS-II-1 or RS-III-1), daily dietary energy intake (kcal/day), education level (primary, secondary general or vocational education, higher vocational education or university), alcohol intake (0, ≤1, >1 glass/day), any vitamins supplement use (yes or no), diabetes (yes or no), hypertension (yes or no) and serum lipid reducing agents use (yes or no);

Effect estimates are based on pooled results of imputed data.

**Supplemental Figure 1.** hPDI-Lifestyle Score and Incident Coronary Heart Disease, Stratified by Sex.


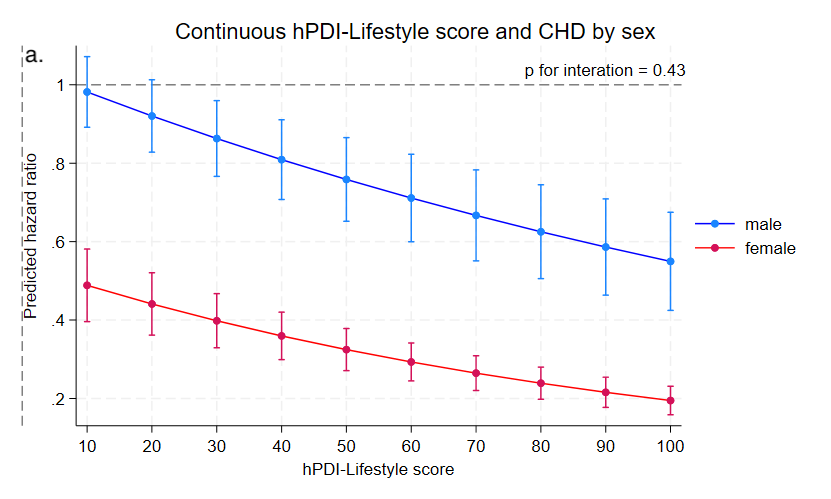


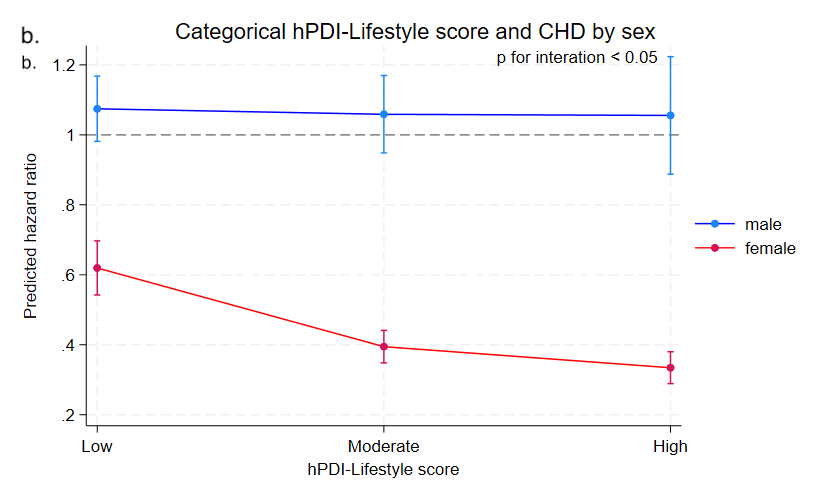


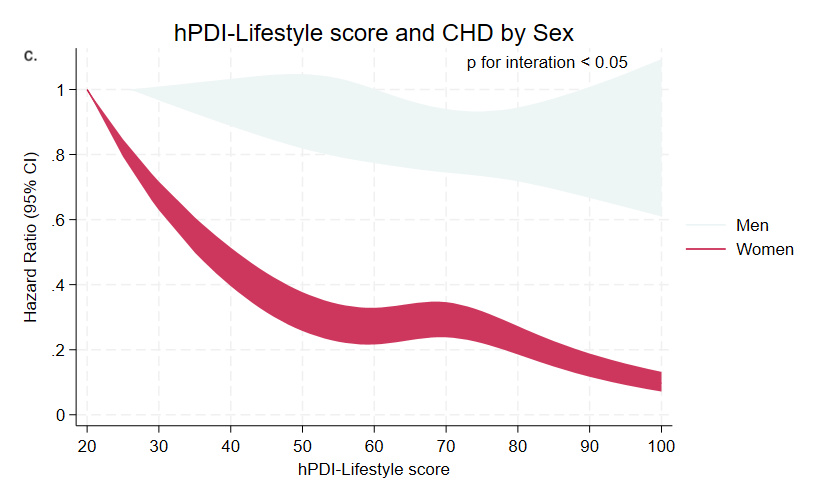


1. Continuous hPDI-Lifestyle Score and Coronary Heart Disease by Sex (Linear Modeling);
2. Categorical hPDI-Lifestyle Score and Coronary Heart Disease by Sex;
3. hPDI-Lifestyle Score and Coronary Heart Disease by Sex (Non-linear Modeling)

Note: The interaction p-values shown in each panel are based on results pooled across 30 imputed datasets, whereas the plots were generated using one randomly selected imputed dataset for visualization. In the categorical model, the inverse association between hPDI-Lifestyle and CHD appeared stronger in women than in men, yielding a nominally significant interaction (*p* < 0·05). In the continuous model, the interaction was not statistically significant (*p* = 0·43). The non-linear spline depiction provides a more flexible visualization of the association, showing wider confidence intervals in men and a slight flattening at higher hPDI-Lifestyle scores, likely reflecting fewer male participants with very high hPDI-lifestyle score. Overall, these patterns suggest that the difference between sexes was modest and not statistically robust across models.

**Supplemental Figure 2.** hPDI-Lifestyle Score and Incident Coronary Heart Disease, Stratified by Genetic Risk.


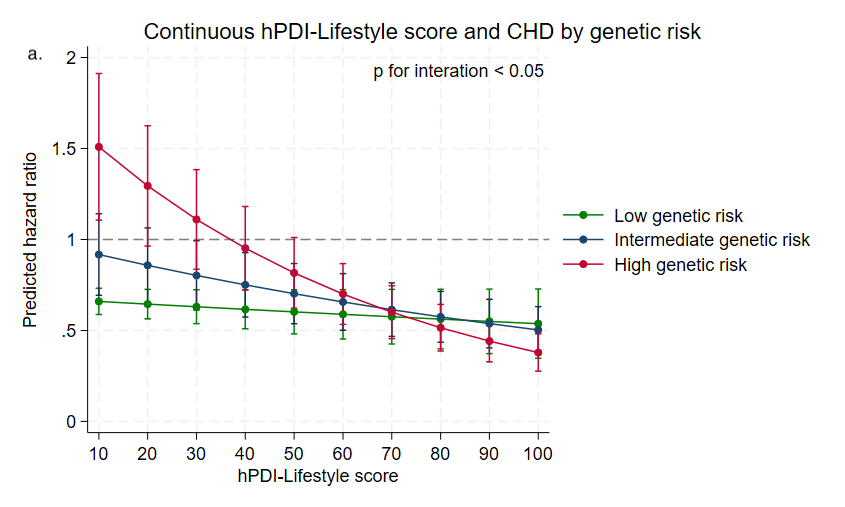


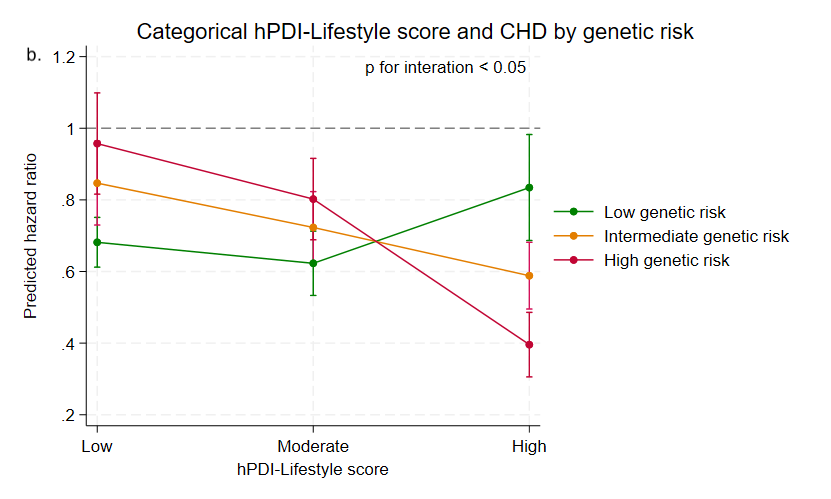


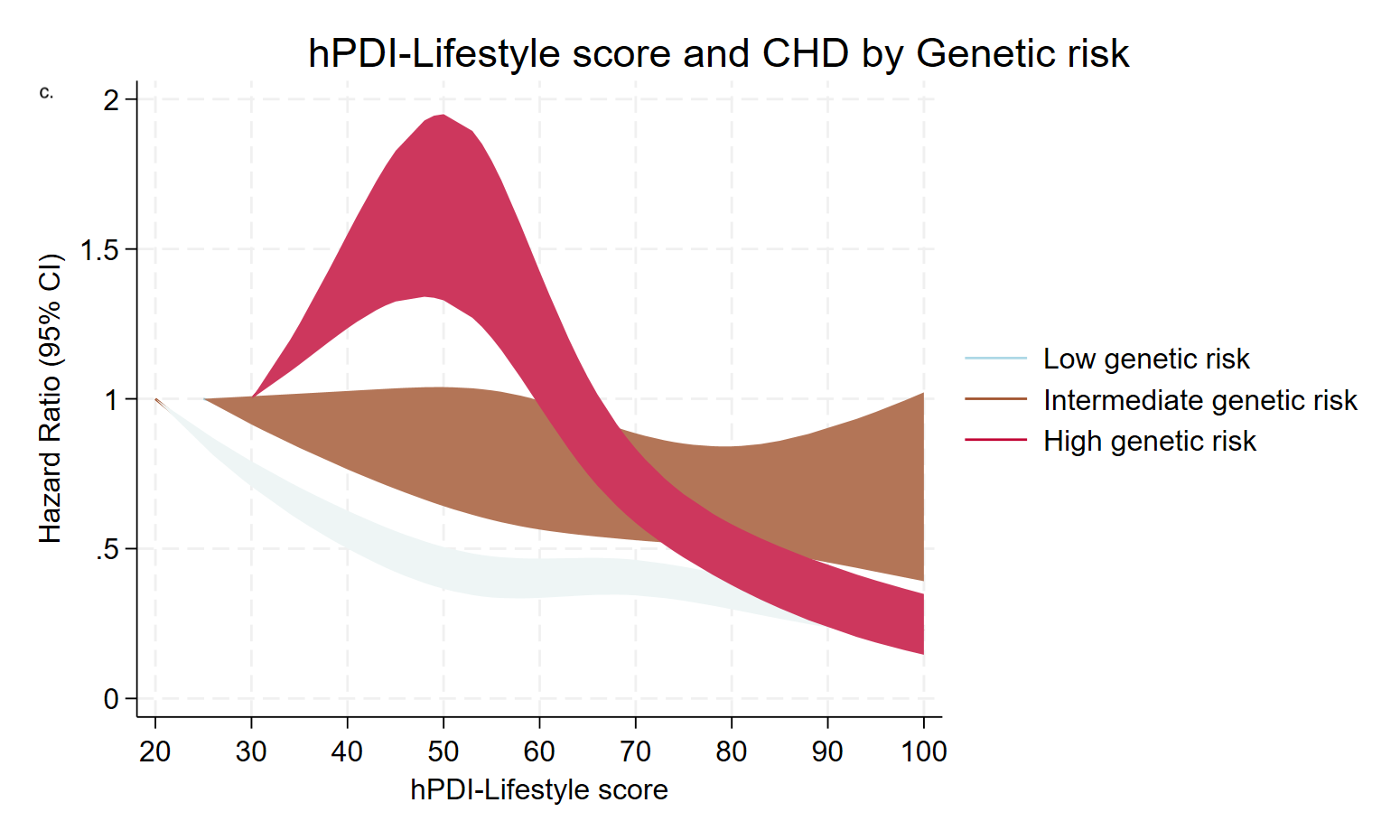


1. Continuous hPDI-Lifestyle Score and Coronary Heart Disease by Genetic Risk (Linear Modeling);
2. Categorical hPDI-Lifestyle Score and Coronary Heart Disease by Genetic Risk;
3. hPDI-Lifestyle Score and Coronary Heart Disease by Genetic Risk (Non-linear Modeling)

Note: The interaction p-values shown in each panel are based on results pooled across 30 imputed datasets, whereas the plots were generated using one randomly selected imputed dataset for visualization. The apparent crossing of hazard ratio lines across genetic risk groups reflects varying slopes of association across genetic-risk strata, and indicates that participants at high genetic risk had greater CHD risk reduction with higher hPDI-Lifestyle adherence which are consistent with the significant interaction reported in the main results.

**Supplemental Figure 3.** Associations of hPDI-Lifestyle Score and Polygenetic Risk Score with the Risk of Incident Conary Heart Disease in the Rotterdam Study Across Different Imputed Datasets.


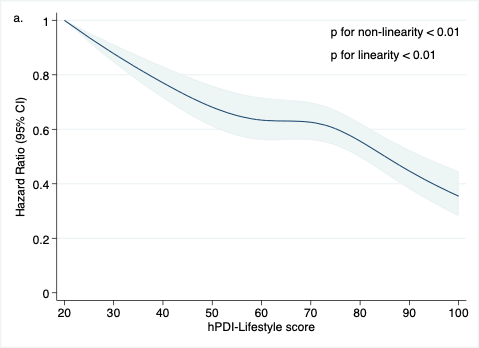


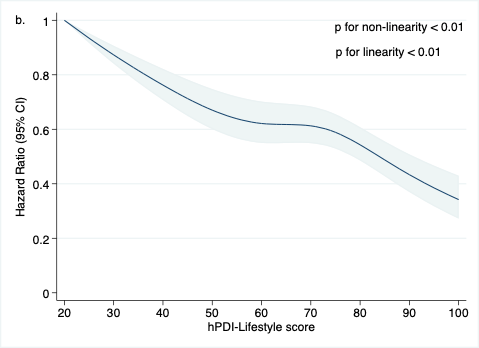


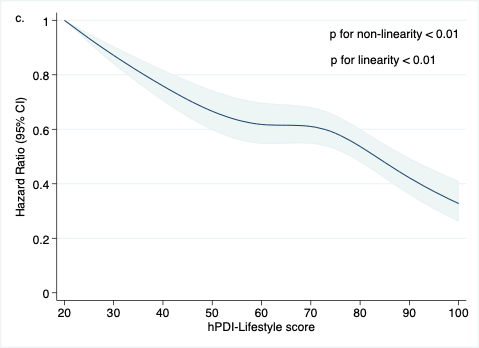


1. hPDI-Lifestyle Score and Incident Coronary Heart Disease (n=9);
2. hPDI-Lifestyle Score and Incident Coronary Heart Disease (n=17);
3. hPDI-Lifestyle Score and Incident Coronary Heart Disease (n=28).

The multivariable-adjusted HRs with 95% CIs of continuous healthy plant-based diet index-lifestyle scores are indicated by the navy blue line and light-blue shading respectively; HRs with 95% CIs were adjusted for sex, age (years old), sub-cohort (RS-I-1, RS-II-1 or RS-III-1), daily dietary energy intake (kcal/day), margarine intake (gram/day), miscellaneous foods intake (gram/day), education level (primary, secondary general or vocational education, higher vocational education or university), alcohol intake (0, ≤1, >1 glass/day), any vitamins supplement use (yes or no), diabetes (yes or no), hypertension (yes or no) and serum lipid reducing agents use (yes or no);

The multivariable-adjusted HRs with 95% CIs of continuous polygenetic risk scores are indicated by the dark line and red shading respectively; HRs with 95% CIs were adjusted for sex and age (years old).
